# Perceptual Integration Compensates for Attention Deficit in Elderly during Repetitive Auditory-Based Sensorimotor Task

**DOI:** 10.3390/s23146420

**Published:** 2023-07-14

**Authors:** Nikita Frolov, Elena Pitsik, Vadim Grubov, Artem Badarin, Vladimir Maksimenko, Alexander Zakharov, Semen Kurkin, Alexander Hramov

**Affiliations:** 1 Laboratory of Dynamics in Biological Systems, Department of Cellular and Molecular Medicine, KU Leuven, 3000 Leuven, Belgium; nikita.frolov@kuleuven.be; 2 Institute of Neuroscience, Samara State Medical University, 443099 Samara, Russia; epitsik@kantiana.ru (E.P.); vgrubov@kantiana.ru (V.G.); badarin.a.a@mail.ru (A.B.); vmaksimenko@kantiana.ru (V.M.); a.v.zaharov@samsmu.ru (A.Z.); aekhramov@kantiana.ru (A.H.)

**Keywords:** EEG, healthy aging, sensorimotor integration, theta rhythm, time-frequency analysis

## Abstract

Sensorimotor integration (SI) brain functions that are vital for everyday life tend to decline in advanced age. At the same time, elderly people preserve a moderate level of neuroplasticity, which allows the brain’s functionality to be maintained and slows down the process of neuronal degradation. Hence, it is important to understand which aspects of SI are modifiable in healthy old age. The current study focuses on an auditory-based SI task and explores: (i) if the repetition of such a task can modify neural activity associated with SI, and (ii) if this effect is different in young and healthy old age. A group of healthy older subjects and young controls underwent an assessment of the whole-brain electroencephalography (EEG) while repetitively executing a motor task cued by the auditory signal. Using EEG spectral power and functional connectivity analyses, we observed a differential age-related modulation of theta activity throughout the repetition of the SI task. Growth of the anterior stimulus-related theta oscillations accompanied by enhanced right-lateralized frontotemporal phase-locking was found in elderly adults. Their young counterparts demonstrated a progressive increase in prestimulus occipital theta power. Our results suggest that the short-term repetition of the auditory-based SI task modulates sensory processing in the elderly. Older participants most likely progressively improve perceptual integration rather than attention-driven processing compared to their younger counterparts.

## 1. Introduction

Healthy aging is accompanied by biological changes in the brain structure and neurochemistry [1,2], such as a reduction in cortical thickness and volume and integrity of white/gray matter. These changes affect cognitive abilities, among which are memory processing, speed, attention [3] and motor performance [4]. In addition, there is evidence that the ability to incorporate the information from afferent inputs and translate it into motor commands tends to decline in advanced age [5,6]. This process is known as sensorimotor integration (SI). It plays a crucial role in an individual’s interaction with the environment, and its age-related impairment negatively influences the quality of the everyday life of elderly people [7].

The current literature recognizes the age-related deficit of the motor cortex excitability as one of the main factors of the reduced functioning of the sensorimotor system. Previous studies probing the intracortical facilitation and inhibition via a transcranial magnetic stimulation (TMS) indicated that the age-related decline in SI is closely related to the impairment of sensory inputs in elderly people [5,8,9]. At the same time, Ferreri et al. revealed that the excessive facilitation of the prefrontal cortex in elderly adults more likely compensated the reduced excitability of the primary motor cortex [10,11]. Along with the reported deficit of motor cortex excitability, physiological aging naturally influences the neural mechanisms of sensory processing. The elderly population experiences difficulties in both uni- and multisensory integration [7,12]. It is well-accepted that the deficit of attention and working memory in the elderly most likely underlies impaired sensory functions [13,14,15,16,17]. In this context, Dushanova and Christov [15] found the age-related distinctions in low-frequency EEG activity, which more likely indicate a decrease in memory operations during sensory processing in elderly adults. Our recent study identified an age-related deficit of working memory as a potential factor in the slowing of auditory-stimulated motor execution [16,18].

Despite the reported declines in cortical excitability, and cognitive and executive functions, early studies evidenced the capability of older people for sensorimotor adaptation [19,20,21,22] and learning complex motor skills [23]. Some studies showed that such abilities decline with age, which can be associated with not only bodily changes but with cognitive aging as well [24]. The review in [25] accumulated substantial evidence of the structural and functional degradation of human brain functionality with age. However, due to a retained, but not high, level of neuroplasticity in healthy old age [26], systematic training invokes compensatory mechanisms to improve sensorimotor functions [21,27]. Several studies reported the modulation of SI in short-term sessions [9,22,28]. For example, Blais et al. demonstrated that preserved perceptual integration improved coordination in elderly subjects under auditory stimulation [22].

The current study aims to complement the existing knowledge on the modulation of brain activity under the repetition of SI tasks in normal aging. Specifically, we consider the data from our previous EEG study on the successive execution of a motor task cued by a pure tone audio signal [16] and evaluate how the neuronal activity associated with SI functions is modified with trial progression in young and healthy old age. We hypothesized that the short-term auditory-based SI session would differentially modulate sensory processing in the considered age groups. As in our previous research, we witnessed a differential age-related involvement of the theta-band oscillations throughout the sensorimotor session, which was dissociated in spatio-temporal domains between the elderly participants and their young counterparts. Our observation could most likely be interpreted as a differential age-related facilitation of the attention- and perception-related mechanisms of sensory processing.

## 2. Materials and Methods

### 2.1. Participants

Thirteen young adults (YA group, 25.5±5.3 SD years, 4F/9M) and eleven elderly adults (EA group, 64.2±7.1 SD years, 4F/7M) were recruited for the study. All subjects were right-handed according to the Edinburgh Handedness Inventory with the minimal score of +70 over both groups. The participants had no medical history of neural pathological conditions, i.e., stroke, head trauma or tumors. All participants signed written informed consent before taking part in the experiments. An experimental study was approved by the local ethics committee of the Immanuel Kant Baltic Federal University and performed in accordance with the Declaration of Helsinki.

### 2.2. Neurophysiological Assessment

During this experiment, the participants were seated in a chair with their hands placed on the armrests, to avoid non-task-related muscle tension. The cortical electrical activity was acquired by EEG recorder Encephalan-EEGR-19/26 (Medicom MTD, Taganrog, Russia) with 31 Ag/AgCl electrodes applied according to the extended 10-20 electrode system site [29]. EEG signals were recorded using 31 sensors {O2, O1, P4, P3, C4, C3, F4, F3, Fp2, Fp1, P8, P7, T8, T7, F8, F7, Oz, Pz, Cz, Fz, Fpz, FT7, FC3, FCz, FC4, FT8, TP7, CP3, CPz, CP4, TP8}. Two reference electrodes A1 and A2 were applied on the earlobes and a ground electrode was placed just above the forehead. The electrodes were placed on the Ten–20 paste (Weaver and Company, Aurora, CO, USA). Acquired EEG signals were sampled at $f_{s}=250$ Hz and presented in the units of μV. The variation of impedance was controlled within the range of 2–5 kΩ during the experiment. The EMG signals were collected from the forearm by the same recording hardware to verify the correctness of the epochs segmentation.

### 2.3. Sensorimotor Integration Session

**Figure 1:**
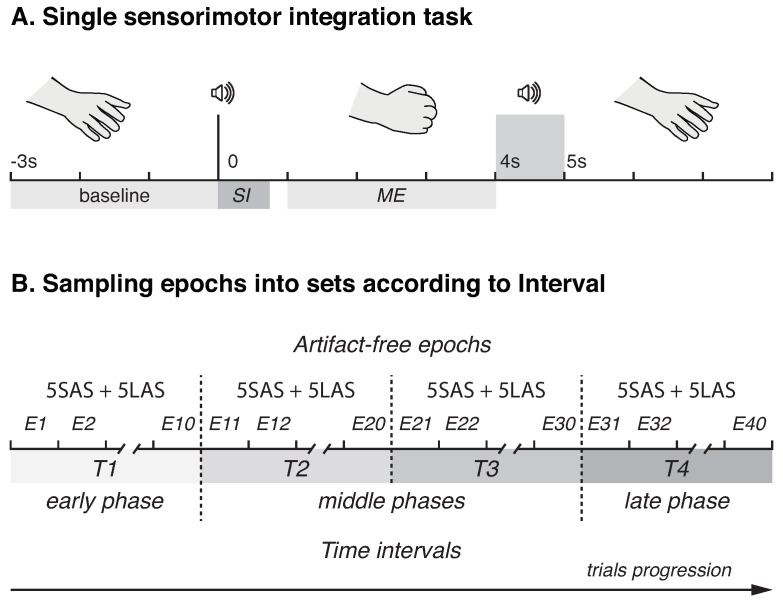
Schematic representation of experimental design. (**A**) A timeline of a single sensorimotor integration task. SI and ME denote sensorimotor integration and motor execution phases, respectively. (**B**) Illustration of sampling EEG epochs according to intervals.

Prior to the experimental session, the details of the procedure were explained to the participants. Each subject underwent the SI session, which consisted of N=60 repetitions of elementary SI task. The task was designed to be simple enough to understand and perform by the participants of advanced age and required the participants to execute movements (ME) cued by auditory signals, pure tone sounds of different duration at 1000 Hz. Specifically, the participant had to clench left or right hand into a fist in response to short (SAS) or long (LAS) audio stimulus, respectively. The durations of SAS and LAS were 300 ms and 750 ms, respectively. The signals were presented at the level of 60 dB via the bilateral speakers located approximately 80 cm in front of the participant. During ME the hand had to be held clenched until the next audio stimulus of the same duration, which informed the participant about the end of ME. To avoid erroneous motor actions, i.e., clenching right hand after SAS or left hand after LAS, we instructed the participants to focus on the correctness of executed actions and accomplish each task at a comfortable speed. The time interval required for SI and ME within single trail was chosen randomly in the range 4–5 s. The pause between the trials was also picked randomly within the range 6–8 s. Figure 1A illustrates a single trial timeline. During the experiment, each participant performed 30 SI task repetitions with each hand. The overall duration of the experimental session was ≈10 min per participant. Experimental session did not include trial runs, but all the participants were given time to familiarize themselves with the sound stimuli prior to the start of the session and provided their subjective confirmation that the stimuli were clearly audible and distinguishable. The same experimental paradigm was previously reported in [16].

### 2.4. EEG Preprocessing and Epochs Segmentation

The raw EEG recordings were filtered using the 50 Hz Notch filter. Additionally, the data were filtered using the 5th-order Butterworth filter in the range 1–100 Hz to remove low-frequency artifacts. The ocular and cardiac artifacts were removed using the independent component analysis (ICA) [30]. The filtered time-series were segmented into 60 epochs 13 s long each according to the experiment protocol. Each epoch included 3 s of prestimulus EEG and 10 s of poststimulus EEG centered at the presentation of the first audio stimulus.

To evaluate the effect of sensorimotor integration task repetition on cortical activation with trial progression, the timeline of experimental session (N=60 epochs total, 30 per stimulus) was divided into four equal intervals: T1 (epochs 1–15); T2 (epochs 16–30); T3 (epochs 31–45); T4 (epochs 46–60). Thus, the interval T1 represented early phase, while the interval T4 represented the last phase of the experiment. The data were then inspected manually and the epochs with the remaining artifacts were rejected. Finally, each interval contained 10 artifact-free epochs (5 epochs per stimulus), 40 total (see Figure 1B for sampling epochs into conditions). In further analyses, all the characteristics of interest were averaged over the epochs within each interval, and the interval was considered a within-subject factor.

The preprocessing steps, including filtering, artifact removal via ICA and epoching, were performed using the MNE package for Python (ver. 0.22.0) [31]. All epochs were baseline-corrected using resting state activity recorded prior to the active phase of the experiment.

### 2.5. Sensor-Level Analysis of Spectral Power

Absolute spectral power (SP) within the range [1,40] Hz and time interval [−3,1.5] s was estimated in sensor space via continuous wavelet transform with Morlet complex-valued mother wavelet [32] and averaged over the epochs in each (group, interval) set. The number of cycles in the wavelet transform was set for each frequency *f* as *f*.

The obtained power spectra were averaged over the distinct frequency bands of interest—theta, lower-1 alpha, lower-2 alpha and upper alpha—closely related to sensory processing and memory. The choice of the frequency bands of interests is due to the results of the previous research using the same dataset, where we demonstrated that, while motor-related effects are mostly accumulated in alpha rhythm [33], the age-related changes in motor initiation phase are most pronounced in theta rhythm, especially in elderly group of subjects [16]. These frequency bands were adjusted individually to each subject using individual alpha peak frequency (IAF) as a peak of alpha frequency within the traditional frequency range based on [34]: theta [IAF−6, IAF−4] Hz; lower-1 alpha [IAF−4, IAF−2] Hz; lower-2 alpha [IAF−2, IAF] Hz; and upper alpha [IAF, IAF+2] Hz. The IAFs and the ranges of corresponding frequency bands are presented in Table 1.

We considered SP changes in two separate time frames:

- *prestimulus activity*: the SP was averaged within the frequency bands of interest over the interval [−2.5,0] s before stimulus presentation and the problem was addressed only in a spatial domain;
- *poststimulus activity*: the SP was considered on the interval [0,1] s after stimulus presentation, i.e., a problem was addressed in both spatial and temporal domains.

To evaluate the interaction of repetition and age, the topograms of differences between the prestimulus SP in the late (T4) and early (T1) phases of experiment were compared between groups via the *F*-test (df1=1, df2=22), and spatial clustering in sensor space was achieved by a non-parametric cluster test with r=2000 random permutations [35]. Similarly, the comparison of poststimulus SP was conducted for (sensor, time) pairs. The cluster-averaged SP were then compared via a mixed-design repeated measures ANOVA with a between-subject factor of age and within-subject factor of interval. The post hoc test aimed at evaluating the difference in the pattern of cluster-averaged SP across intervals for different age groups, i.e., T4 vs. T1 for YA and T4 vs. T1 for EA, was conducted via a dependent *t*-test (df=22) or Wilcoxon signed-rank test. The within-individual temporal associations of cluster-averaged SP versus intervals {T1, T2, T3, T4} in considered age groups were yielded by the repeated measures correlation (RM corr) analysis [36].

Calculation of power spectra and cluster-based statistics was performed via the toolboxes for time–frequency and statistical analyses implemented in MNE package for Python (ver. 0.22.0). Mixed-design repeated measures ANOVA and post hoc tests were performed using JASP software. RM corr analysis and its visualization were conducted using pingouin package for Python (ver. 0.3.10) [37].

### 2.6. Sensor-Level Connectivity Analysis

To support the results of spectral power analysis, we explored the reconfiguration of functional connectivity [38] within the same time frames and frequency bands, where SP exhibited significant effect of repetition and age. We evaluated functional connectivity in terms of phase synchrony and used phase locking value, PLV, as a proper metric [39]. To calculate PLV for a pair of sensors *x* and *y* we used a frequency domain definition [40]:

(1) $$PLV=|E[S_{xy}/|S_{xy}|]|,$$

where E[•] determined averaging over epochs and $S_{xy}$ denoted cross-spectral density of sensors *x* and *y* within the frequency band of interest. Thus, iterating over all possible pairs of sensors, we obtained 31×31 connectivity matrices for each (group, interval) set filled with values of PLV averaged over the epochs for each subject.

To evaluate the interaction of repetition and age on functional connectivity, the matrices composed of PLV differences in the late (T4) and early (T1) stages of the experiment were compared between groups using Network-Based Statistics approach, NBS [41]. Briefly, NBS is a non-parametrical statistical test aimed at inference of closed subnetworks exhibiting significant change in functional connectivity measure between experimental groups or conditions. The values of PLV averaged over the observed subnetworks were then compared via a mixed-design repeated measures ANOVA with a between-subject factor of age and within-subject factor of interval. The post hoc test aimed at evaluating the difference in the pattern of averaged PLV across intervals for different age groups, i.e., T4 vs. T1 for YA and T4 vs. T1 for EA, was conducted via a dependent *t*-test (df=22). The within-individual temporal associations of averaged PLV versus intervals {T1, T2, T3, T4} in considered age groups were yielded by the repeated measures correlation (rm corr) analysis.

Calculation of functional connectivity was performed via the toolbox for connectivity analysis implemented in MNE package for Python (ver. 0.22.0).

## 3. Results

### 3.1. Effect of Repetition and Age on the Prestimulus Spectral Power

**Figure 2:**
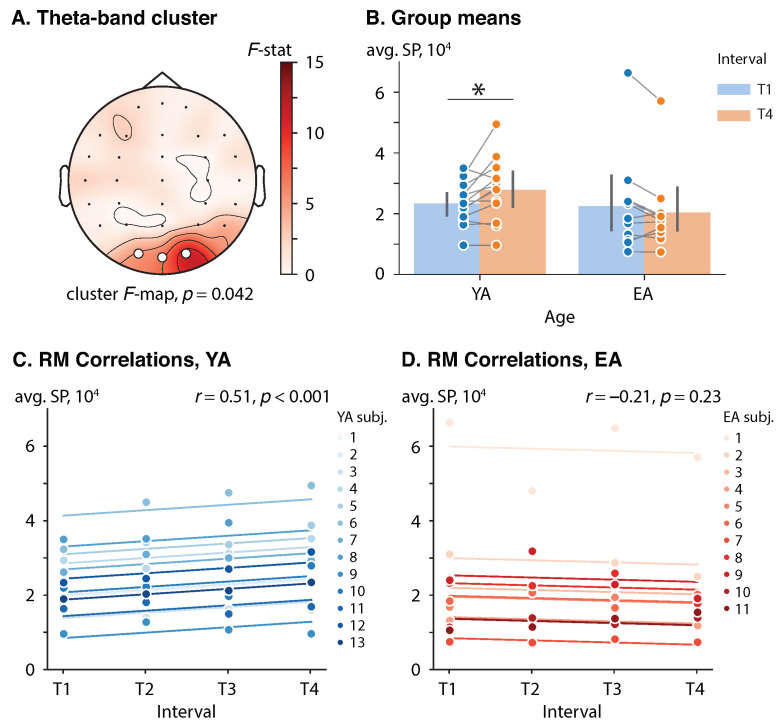
Effect of repetition and age on the prestimulus SP. (**A**) Cluster *F*-maps of the prestimulus theta-band SP difference (T4 vs. T1). White circles indicate EEG sensors comprising the significant cluster. (**B**) Group means with one standard deviation of the prestimulus theta-band cluster-averaged SP. Blue and orange circles show cluster-averaged SP for each subject in each (group, interval) pair. Here, ‘*’ indicates the level of significance p<0.05. (**C**,**D**) Rmcorr plots of the prestimulus theta-band SP versus interval for YA and EA groups, respectively. Observations from each subject are presented in the same color with corresponding line indicating the rmcorr fit for each subject. Rmcorr coefficients *r* and corresponding *p*-values are shown in the panels.

In the prestimulus interval [−2.5,0] s, a significant cluster-level effect of age on the SP difference (T4 vs. T1) was found in the theta frequency band ($\alpha_{cl}=0.05$, $F_{cl}=4.30$). The theta-band cluster was comprised of the occipital EEG sensors {O2, Oz, O1} (p=0.042). See Figure 2A for the cluster *F*-map. A mixed-design ANOVA did not show a significant effect of either age $(F_{\left(1,22\right)}=0.77$, p=0.39, $\eta_{p}^{2}=0.03)$ or interval $(F_{\left(1,22\right)}=1.28$, p=0.25, $\eta_{p}^{2}=0.06)$ on the prestimulus cluster-averaged SP in considered (group, interval) pairs. However, the interaction of these factors was significant $(F_{\left(1,22\right)}=9.54$, p=0.005, $\eta_{p}^{2}=0.30)$.

Each groups’ cluster-averaged SPs in the intervals T1 and T4 were analyzed via a dependent *t*-test with Bonferroni correction to reveal how the brain adjusts its prestimulus theta-band activation in the course of the experiment in different groups. The YA subjects demonstrated a 4.3 × 10${}^{3}$ units increase in the cluster-averaged SP from early phase (T1) to late phase (T4), $t_{\left(12\right)}=2.82$, p=0.016, d=0.78. In turn, in the EA subjects, the difference in the cluster-averaged SP between the T4 and T1 intervals was not significant, $t_{\left(10\right)}=1.55$, p=0.15, d=0.47. See Figure 2B for the group means.

Additionally, in the YA group, a prestimulus cluster-averaged SP was positively correlated with interval, $r_{\left(38\right)}=0.51$, p<0.001, which implied a consistent change in the prestimulus theta-band SP in occipital sensors throughout the session. Conversely, no correlation between the prestimulus cluster-averaged SP and interval was observed in the EA group, $r_{\left(32\right)}=-0.21$, p=0.23. See Figure 2C,D for the rmcorr plots for the YA and EA groups, respectively.

No effect of age and interval was found in the lower-1, lower-2, and upper alpha bands.

### 3.2. Effect of Repetition and Age on the Poststimulus Spectral Power

**Figure 3:**
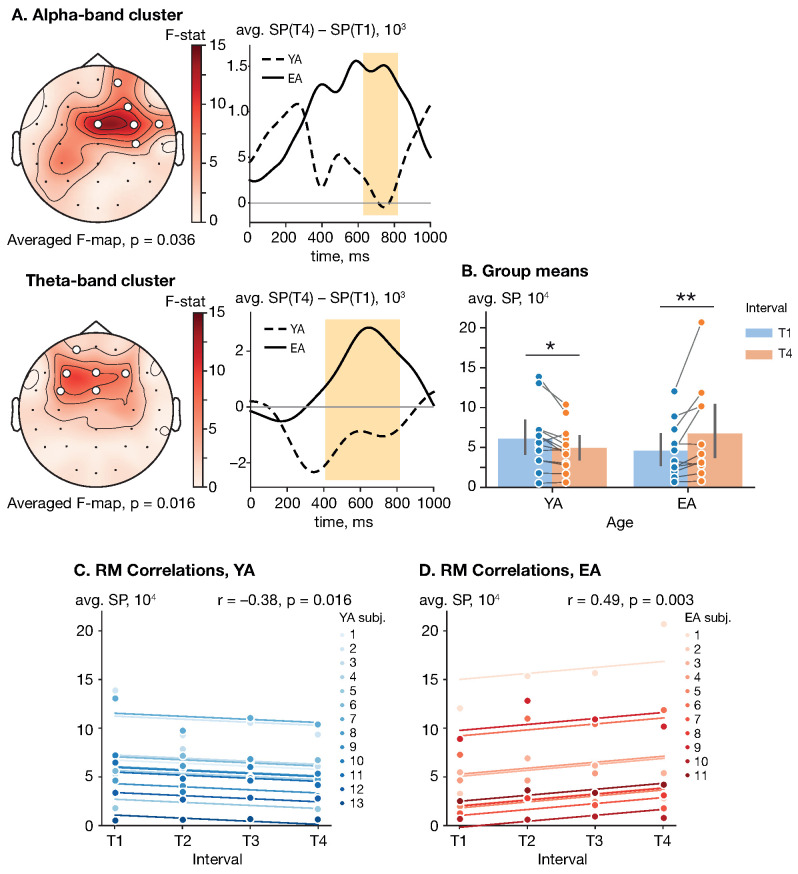
Effect of repetition and age on the poststimulus SP. (**A**) Cluster *F*-maps of the poststimulus alpha- and theta-band SP difference (T4 vs. T1). White circles indicate EEG sensors comprising the significant cluster. (**B**) Group means with one standard deviation of the poststimulus theta-band cluster-averaged SP. Blue and orange circles show cluster-averaged SP for each subject in each (group, interval) pair. Here, ‘*’ indicates the level of significance p<0.05, and ‘**’ indicates the level of significance p<0.01. (**C**,**D**) Rmcorr plots of the poststimulus theta-band SP versus interval for YA and EA groups, respectively. Observations from each subject are presented in the same color with corresponding line indicating the rmcorr fit for each subject. Rmcorr coefficients *r* and corresponding *p*-values are shown in the panels.

In the poststimulus interval related to sensory processing [0,1] s, a significant cluster-level effect of age on the SP difference (T4 vs. T1) was observed in both the alpha and theta frequency bands ($\alpha_{cl}=0.015$, $F_{cl}=6.96$). The poststimulus alpha cluster appeared at 632–816 ms and included right-lateralized frontal, central and temporal sensors {Fp2, F4, FC4, FCz, FT8, C4} (p=0.036). The poststimulus theta-band cluster appeared at 412–812 ms and included left-lateralized frontal and frontocentral electrodes {FC3, FCz, F3, Fz, F4, Fp1} (p=0.016). See Figure 3A for the cluster *F*-map. Regarding the cluster-averaged SP, neither age $(F_{\left(1,22\right)}=0.01$, p=0.92, $\eta_{p}^{2}=4.7*10^{-4})$ nor interval $(F_{\left(1,22\right)}=1.22$, p=0.28, $\eta_{p}^{2}=0.05)$ had a significant effect as shown by a mixed-design ANOVA. However, the interaction between these factors significantly affected the poststimulus theta-band SP ($F_{\left(1,22\right)}=12.79$, p=0.002, $\eta_{p}^{2}=0.37$).

To explore the interaction effect in detail, each groups’ cluster-averaged SPs on the intervals T1 and T4 were analyzed via a dependent *t*-test (YA) and Wilcoxon signed-rank test (EA) with Bonferroni correction. The YA group demonstrated a decrease in the cluster-averaged poststimulus SP from the early phase ($M=6.07*10^{4}$, $SD=3.87*10^{4}$) to the late phase ($M=4.94*10^{4}$, $SD=2.77*10^{4}$) with $t_{\left(12\right)}=-2.75$, p=0.018 and d=−0.76. Conversely, the EA subjects showed an increase in the cluster-averaged SP from the early phase ($M=4.60*10^{4}$, $SD=3.54*10^{4}$) to the late phase ($M=6.74*10^{4}$, $SD=5.95*10^{4}$) with $W_{\left(10\right)}=4.0$, p=0.007 and rank-biserial correlation =−0.88. See Figure 3B for the group means.

The RM correlation analysis revealed that the cluster-averaged SP was positively correlated with interval in the EA group, $r_{\left(32\right)}=0.49$, p=0.003. At the same time, YA subjects demonstrated an opposite significant trend, $r_{\left(38\right)}=-0.38$, p=0.016. See Figure 3C,D for the rmcorr plots for the YA and EA groups, respectively.

No effect of age and interval was found in the lower-1, lower-2 and upper alpha bands.

### 3.3. Effect of Repetition and Age on Functional Connectivity

**Figure 4:**
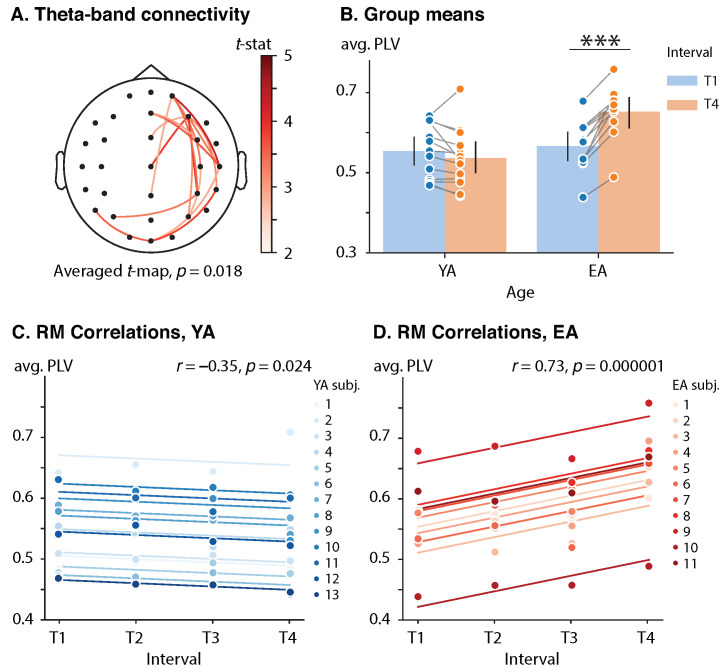
Effect of repetition and age on the poststimulus connectivity. (**A**) Cluster of the poststimulus theta-band connectivity difference (T4 vs. T1). Functional links are color-coded by the value of unpaired *t*-statistic. (**B**) Group means with one standard deviation of the poststimulus theta-band cluster-averaged PLV. Blue and orange circles show cluster-averaged PLV for each subject in each (group, interval) pair. Here, ‘***’ indicates the level of significance p<0.001. (**C**,**D**) Rmcorr plots of the poststimulus theta-band PLV versus interval for YA and EA groups, respectively. Observations from each subject are presented in the same color with corresponding line indicating the rmcorr fit for each subject. Rmcorr coefficients *r* and corresponding *p*-values are shown in the panels.

A significant effect of age on the theta-band PLV difference (T4 vs. T1) was only observed in the poststimulus time frame 412–812 ms ($\alpha_{cl}=0.005$, $t_{cl}=2.819$). The functional subnetwork involved interactions between midline sensors, right-lateralized frontal, temporal, parietal and occipital sensors, and left-lateralized parietal sensors (p=0.018). See Figure 4A for the subnetwork visualization. The mixed designed ANOVA indicated a significant effect of both age ($F_{\left(1,22\right)}=5.674$, p=0.026, $\eta^{2}=0.164$) and interval ($F_{\left(1,22\right)}=21.563$, p<0.001, $\eta^{2}=0.046$) on PLV averaged over the subnetwork. However, the most notable was the significance of the interaction between those factors ($F_{\left(1,22\right)}=49.777$, p<0.001, $\eta^{2}=0.106$).

Each group’s averaged values of PLV were compared between the T4 and T1 conditions via a dependent *t*-test. The YA group did not demonstrate a significant change in the averaged PLV between the T1 (M=0.554, SD=0.067) and T4 (M=0.536, SD=0.074) conditions with $t_{\left(12\right)}=-1.809$, p=0.096 and d=−0.502. At the same time, the EA participants exhibited a significant growth in the poststimulus theta-band coupling from the early (M=0.566, SD=0.064) and late (M=0.652, SD=0.067) stages of the experiment with $t_{\left(10\right)}=7.806$, p<0.001 and d=2.354. See Figure 4B for the group means.

The RM correlation analysis revealed that the value of PLV averaged over the poststimulus theta-band functional network was positively correlated with interval in the EA group, $r_{\left(32\right)}=0.73$, p<0.001. At the same time, YA subjects demonstrated an opposite significant trend, $r_{\left(38\right)}=-0.35$, p=0.024. See Figure 4B,C for the rmcorr plots for the YA and EA groups, respectively.

## 4. Discussion

We used EEG to explore how the repetition of an auditory-based SI task affected cortical activation in healthy elderly people and their young counterparts. We found that in young subjects, prestimulus occipital theta band power increases with the sensorimotor trial’s progression. In elderly adults, we observed the modulation of stimulus-related frontal theta oscillations throughout the experimental session. Notably, this modulation in elderly adults was accompanied by the enhancement of the phase-locking of frontotemporal and frontoparietal interactions. We discuss our findings further in light of the potential mechanisms underlying the observed changes.

We can conclude from these observations that the repeated sensorimotor task caused differential age-related modulation of theta-band activity. Young subjects demonstrated a significant growth in prestimulus occipital theta SP and attenuation of stimulus-related frontal theta oscillations with the trials’ progression. Elderly participants showed a reverse effect, in which the increase in anterior theta SP was accompanied by enhanced frontotemporal phase synchrony in the right hemisphere.

The alpha-band cluster observed in the right-lateralized central, frontal and temporal lobes is most likely associated with motor-related desynchronization of the alpha rhythm during motor execution. The age-related differences in motor-related activity were previously described in [16,42], and are not discussed in the present research.

We observed the increased prestimulus theta power in the occipital EEG sensors overlapping the visual cortex, which increased significantly in the YA group but not in EA. Event-related synchronization in theta activity is known to reflect the cognitive processes [43], and occipital theta power can even be used as a diagnostic test for the prediction of neurodegeneration with a sensitivity of 79% and specificity of 77% [44]. The authors of [45] used a memory retrieval task to demonstrate that an increase in prestimulus theta activity can be associated with better source memory accuracy. Another research [46] showed high posterior theta-band power occurring in the time interval between the cue and the formation of ERD in a motor imagery task, which the authors hypothesized was related to the cue processing. The authors also linked this observation to a widely reported negative correlation between theta-band power and default mode network (DMN) activation [47]. However, this effect is commonly found in the frontal lobe, whereas we reported theta activation in the occipital cortex, which is widely reported to be associated with attention. Specifically, an increase in attention is associated with a decrease in theta-band power [48]. The observed increase in prestimulus theta-band power throughout the experiment can be associated with working memory consolidation, as a motor-related memory consolidation task based on neurofeedback training has previously reported its positive influence on theta-band power [49].

Unlike their young counterparts, elderly subjects demonstrated an enhanced stimulus-related modulation of theta-band activity. Firstly, observed activation of the frontal areas in the early stages of sensory processing (see Figure 3) most likely indicates executive control and access to working memory [50]. Previous studies reported that the exact role of the frontal cortex in working memory is manipulating information streams to posterior brain regions [51,52]. In this regard, our results are mainly consistent with a previous study by Kawasaki et al., which showed a similar oscillatory pattern in the frontal area during the auditory working memory task [53]. The authors concluded that the modulation of theta oscillations in the frontal cortex distinguishes the executive functions of working memory from the maintenance of sensory information as short-term mental representations. Notably, more prominent engagement of frontal areas in elderly participants during auditory processing is not surprising due to the well-accepted frontal shift in aging, which is supposed to be a compensatory mechanism to confront neural decline [54]. Secondly, right-lateralized frontotemporal interaction showed greater increases in the elderly group throughout the repetition of the sensorimotor task (see Figure 4). Taking into account the fact that the task required distinguishing between durations of pure tone stimuli, this result is consistent with past studies indicating right-hemispheric dominance of the auditory cortex in the perception of non-speech auditory stimuli and slow spectral modulations [55,56]. The revealed connectivity pattern may be considered an enhancement of the dorsal auditory processing stream, which involves interaction between the auditory and motor systems [57,58]. Finally, the observed age-related asymmetry in neuronal processing and overall loss of left-hemispheric dominance may be associated with the rightward white matter distribution under the auditory cortex in the older population [59,60].

While our study considers the changes found in healthy aging elderly people, we should mention the changes found in various neurodegenerative diseases, such as Alzheimer (AD) and Parkinson’s (PD) disease, which is a variant of pathological aging. According to Reference [61], compared with healthy people, changes were observed in the left parietal, occipital, right frontal and temporal brain regions using EEGs, magnetoencephalography (MEGs) and functional magnetic resonance imaging (fMRI). It may be considered a potential biomarker of functional impairment (AD). Moreover, there is an increase in entropy in the frontal, central, parietal, occipital and temporal brain regions in Parkinson’s disease [62]. The observed changes in the group of elderly adults can therefore be considered as universal changes in the activity of the neural networks of the brain, whose dysfunction is significantly exacerbated in pathology. The discovered frontotemporal connectivity during the processing of an auditory stimulus in the context of the performance of a motor task is the rationale for the effectiveness of rehabilitation techniques (using audio cuing and music therapy) in reducing the severity of motor disorders in PD [63].

## 5. Conclusions

Repetition of a sensorimotor integration task differentially affected theta-band activation in young and elderly adult groups. Young subjects demonstrated an increase in prestimulus theta-band spectral power over posterior EEG sensors, also known as auditory occipital activation, throughout the session. Elderly adults exhibited a progressive stimulus-related engagement of anterior brain areas accompanied by strengthening frontotemporal interactions in the right hemisphere. Our findings suggest that in elderly adults, an auditory-based sensorimotor session facilitates perceptual integration, which most probably aims to compensate the age-related deficit of attention. The presented results may potentially contribute to the field of mild neurological impairments’ diagnostics, but these perspectives need further extended investigation.

Our research is not without limitations. We consider the flaws in the experimental design as the most notable of these. Despite the fact that the results of this and the previous research are in line with the well-known concepts presented in the scientific literature, we are aware that the chosen stimulation model (lack of focus phase, different duration of audio stimuli, etc.) can potentially affect the results of neurophysiological measurements. Therefore, the research paradigm of this study requires further verification on other motor-related EEG datasets.

## Figures and Tables

**Table 1:** Individual alpha frequency (IAF) and individually defined frequency bands.

Group, No.	IAF	Theta	Lower-1 Alpha	Lower-2 Alpha	Upper Alpha
YA, 1	10.4 Hz	4.4–6.4 Hz	6.4–8.4 Hz	8.4–10.4 Hz	10.4–12.4 Hz
YA, 2	11.0 Hz	5.0–7.0 Hz	7.0–9.0 Hz	9.0–11.0 Hz	11.0–13.0 Hz
YA, 3	10.3 Hz	4.3–6.3 Hz	6.3–8.3 Hz	8.3–10.3 Hz	10.3–12.3 Hz
YA, 4	10.5 Hz	4.5–6.5 Hz	6.5–8.5 Hz	8.5–10.5 Hz	10.5–12.5 Hz
YA, 5	9.4 Hz	3.4–5.4 Hz	5.4–7.4 Hz	7.4–9.4 Hz	9.4–11.4 Hz
YA, 6	9.4 Hz	3.4–5.4 Hz	5.4–7.4 Hz	7.4–9.4 Hz	9.4–11.4 Hz
YA, 7	10.1 Hz	4.1–6.1 Hz	6.1–8.1 Hz	8.1–10.1 Hz	10.1–12.1 Hz
YA, 8	9.0 Hz	3.0–5.0 Hz	5.0–7.0 Hz	7.0–9.0 Hz	9.0–11.0 Hz
YA, 9	10.5 Hz	4.5–6.5 Hz	6.5–8.5 Hz	8.5–10.5 Hz	10.5–12.5 Hz
YA, 10	10.9 Hz	4.9–6.9 Hz	6.9–8.9 Hz	8.9–10.9 Hz	10.9–12.9 Hz
YA, 11	10.8 Hz	4.8–6.8 Hz	6.8–8.8 Hz	8.8–10.8 Hz	10.8–12.8 Hz
YA, 12	9.5 Hz	3.5–5.5 Hz	5.5–7.5 Hz	7.5–9.5 Hz	9.5–11.5 Hz
YA, 13	10.6 Hz	4.6–6.6 Hz	6.6–8.6 Hz	8.6–10.6 Hz	10.6–12.6 Hz
EA, 1	8.9 Hz	2.9–4.9 Hz	4.9–6.9 Hz	6.9–8.9 Hz	8.9–10.9 Hz
EA, 2	10.3 Hz	4.3–6.3 Hz	6.3–8.3 Hz	8.3–10.3 Hz	10.3–12.3 Hz
EA, 3	8.7 Hz	2.7–4.7 Hz	4.7–6.7 Hz	6.7–8.7 Hz	8.7–10.7 Hz
EA, 4	8.5 Hz	2.5–4.5 Hz	4.5–6.5 Hz	6.5–8.5 Hz	8.5–10.5 Hz
EA, 5	10.0 Hz	4.0–6.0 Hz	6.0–8.0 Hz	8.0–10.0 Hz	10.0–12.0 Hz
EA, 6	9.1 Hz	3.1–5.1 Hz	5.1–7.1 Hz	7.1–9.1 Hz	9.1–11.1 Hz
EA, 7	10.4 Hz	4.4–6.4 Hz	6.4–8.4 Hz	8.4–10.4 Hz	10.4–12.4 Hz
EA, 8	11.5 Hz	5.5–7.5 Hz	7.5–9.5 Hz	9.5–11.5 Hz	11.5–13.5 Hz
EA, 9	9.1 Hz	3.1–5.1 Hz	5.1–7.1 Hz	7.1–9.1 Hz	9.1–11.1 Hz
EA, 10	11.1 Hz	5.1–7.1 Hz	7.1–9.1 Hz	9.1–11.1 Hz	11.1–13.1 Hz
EA, 11	10.1 Hz	4.1–6.1 Hz	6.1–8.1 Hz	8.1–10.1 Hz	10.1–12.1 Hz

## Data Availability

The datasets presented in this study can be found at dx.doi.org/10.6084/m9.figshare.14695578.v1 (accessed on 12 July 2023).
